# Quantitative ultrasound imaging of therapy response in bladder cancer *in vivo*

**DOI:** 10.18632/oncoscience.302

**Published:** 2016-04-18

**Authors:** William T. Tran, Lakshmanan Sannachi, Naum Papanicolau, Hadi Tadayyon, Azza Al Mahrouki, Ahmed El Kaffas, Alborz Gorjizadeh, Justin Lee, Gregory J. Czarnota

**Affiliations:** ^1^ Sunnybrook Health Sciences Centre, Department of Radiation Oncology, Toronto Canada; ^2^ Sheffield Hallam University, Centre for Health and Social Care Research, Sheffield UK; ^3^ University of Toronto, Department of Medical Biophysics, Toronto Canada; ^4^ Ryerson University, Department of Computer Science, Toronto Canada; ^5^ University of Toronto, Department of Radiation Oncology, Toronto Canada

**Keywords:** quantitative ultrasound, ultrasound, vascular disrupting agents, radiation therapy

## Abstract

**Background and Aims:**

Quantitative ultrasound (QUS) was investigated to monitor bladder cancer treatment response *in vivo* and to evaluate tumor cell death from combined treatments using ultrasound-stimulated microbubbles and radiation therapy.

**Methods:**

Tumor-bearing mice (n=45), with bladder cancer xenografts (HT- 1376) were exposed to 9 treatment conditions consisting of variable concentrations of ultrasound-stimulated *Definity* microbubbles [nil, low (1%), high (3%)], combined with single fractionated doses of radiation (0 Gy, 2 Gy, 8 Gy). High frequency (25 MHz) ultrasound was used to collect the raw radiofrequency (RF) data of the backscatter signal from tumors prior to, and 24 hours after treatment in order to obtain QUS parameters. The calculated QUS spectral parameters included the mid-band fit (MBF), and 0-MHz intercept (SI) using a linear regression analysis of the normalized power spectrum.

**Results and Conclusions:**

There were maximal increases in QUS parameters following treatments with high concentration microbubbles combined with 8 Gy radiation: (ΔMBF = +6.41 ± 1.40 (±SD) dBr and SI= + 7.01 ± 1.20 (±SD) dBr. Histological data revealed increased cell death, and a reduction in nuclear size with treatments, which was mirrored by changes in quantitative ultrasound parameters. QUS demonstrated markers to detect treatment effects in bladder tumors *in vivo*.

## INTRODUCTION

Worldwide, approximately 385,000 cases of bladder cancer are diagnosed annually [1]. For localized muscle-invasive bladder cancer (i.e., T≥1, N0, M0) treatment will typically involve cystectomy with urinary diversion. Recently however, neoadjuvant chemotherapy followed by definitive surgery has been shown to improve survival by 5-8%, for clinically operable and muscle-invasive bladder cancer [2]. Other strategies, such as pre-operative radiotherapy has demonstrated improved local control for T3b tumors, but a consensus on survival benefits using pre-operative radiotherapy followed by cystectomy still remains unclear [2]. The overall survival is dependent on tumor size, invaseness to adjacent parenchyma and nodal status; 5-year survival rates are approximately 50% and decrease to 20-40% when there is nodal involvement [3].

In recent years, studies have examined concomitant and multimodality treatments to improve survival outcomes. In a phase III, multicenter study, James *et al.* showed greater locoregional control using synchronous chemoradiation, which combined pyrimidine analogs such as fluorouracil, and other cytotoxic agents like mitomycin C, and fractionated radiation [1]. The study examined 360 patients with muscle-invasive bladder cancer and found an overall 2-year disease-free survival of 67% within the chemoradiation arm, compared to 54% in patients who received radiotherapy alone [1]. Clinical benefits also included equivalent reporting of adverse events; suggesting also that combinatory treatments could potentiate the therapeutic ratio [1]. Indeed, concurrent radiotherapy with other treatment modalities is attractive- if additive effects are achieved, while mitigating adverse events. Such a therapeutic paradigm has motivated studies using radiotherapy and antivascular agents, such as 5,6-dimethylxanthenone-4-acetic acid (DMXAA) concomitantly [4]. Pre-clinical data by Wilson *et al*. have shown that additive effects were achieved when daily, fractionated radiotherapy was combined with DMXAA, resulting in an overall tumor growth delay in tumor-bearing mice [4]. Vascular disrupting agents have been the focus of several ongoing studies and recently, we reported using combined radiotherapy and ultrasound mediated microbubbles as an antivascular agent in bladder cancer xenografts [5]. Earlier findings from that study have indicated that ultrasound-mediated microbubbles can enhance radiotherapy effects and decrease vascular density. Concomitant treatments using radiation and microbubbles were shown to cause concurrent biological responses that involve both tumor cells and endothelial cells of the tumor vasculature [5, 6].

Microbubbles are gas-filled microspheres composed of a biopolymer, protein or lipid shell and traditionally used for contrast enhancement in vascular sonography. When exposed to an acoustic field, microbubbles demonstrate good echogenicity because of an increase in scatterers, and from preferential harmonic detection. However, at higher acoustic pressures, ultrasound can cause microbubble disruption through cavitation and oscillations. Previous studies have shown that when bubbles are introduced to the tumor vasculature and insonified using high mechanical indices, the mechanical forces can perturb endothelial cells within the vessels themselves [7]. There is evidence to support that this disruption initiates cell death signalling similar to radiation response mechanisms, and can lead to additive tumor damage [8]. Traditionally, evaluating these treatment effects are performed using gold-standard immunohistochemical techniques on the pathological specimens. However, there is evidence to suggest that quantitative ultrasound imaging can also provide biomarkers to characterize tissue features in response to therapy [9-11].

### Tissue characterization using quantitative ultrasound

Although ultrasound imaging has been developed for decades, QUS techniques for tissue characterization are relatively newer in comparison. QUS studies were significantly advancing in the 1980s from seminal works by Lizzi *et al*., [12, 13], Feleppa *et al*., [14] and later studies by Insana & Hall [15]. QUS analysis uses either high or low frequency ultrasound information collected from tissue at both cellular and subcellular levels. In contrast to conventional b-mode ultrasound, QUS retains the digital radiofrequency (RF) echo signals that are typically discarded in grey-scale sonography. In retaining the digital RF information, signal processing involves applying a gated Hamming function within a discrete line segment of the RF signal along the axial direction. A frequency-dependent power spectrum is subsequently computed by using a Fourier transform of the signal. However due to system and transducer artefacts that can modulate the power spectrum; a correction is applied using a calibration pulse and the resulting normalized power spectrum is generated and denoted in units as *dBr* [16]. The calibration pulse is typically obtained from a tissue-mimicking phantom made from agar-embedded glass microspheres with known acoustic properties such as scatterer size, concentration, speed of sound, backscatter coefficient, and attenuation coefficient [17]. Lizzi *et al*. previously studied changes in tissue scattering characteristics using spectrum analysis. QUS parameters such as the mid-band fit (MBF) and 0-MHz intercept (SI), from the power spectrum were shown to reflect microstructural changes caused by cell death, in ocular tumors treated with hyperthermia [18]. Findings from Lizzi *et al.* were later adapted by Czarnota *et al*., and Kolios *et al*., to study chemotherapy-driven cell death mechanisms such as apoptosis *in vitro* and *in vivo* using QUS [19, 20]. These studies examined the ultrasound backscatter of acute myeloid leukemia before and after exposure to Cisplatin. Structural alterations such as, pyknosis and karyorhexis caused by chemotherapyinduced cell death were observed following treatment and showed an increase in the backscatter intensity, as early as 24 hours after treatment [19, 20]. These QUS techniques have since been demonstrated to be effective in monitoring a variety of tumor models including skin, head and neck, prostate, breast, and acute myeloid leukemia [7, 10, 11, 21-23]. In the present study, we apply QUS techniques to investigate treatment-induced cell death in bladder cancer xenografts and build on our previous report using ultrasound-stimulated microbubbles and radiation *in vivo* [5]. Here, 45 tumor-bearing mice were treated with combined ultrasound-stimulated microbubbles and radiation, and tumors were imaged using ultrasound before treatment, and then after 24 hours to evaluate treatment effects using spectrum analysis.

## RESULTS

**Figure 1:**
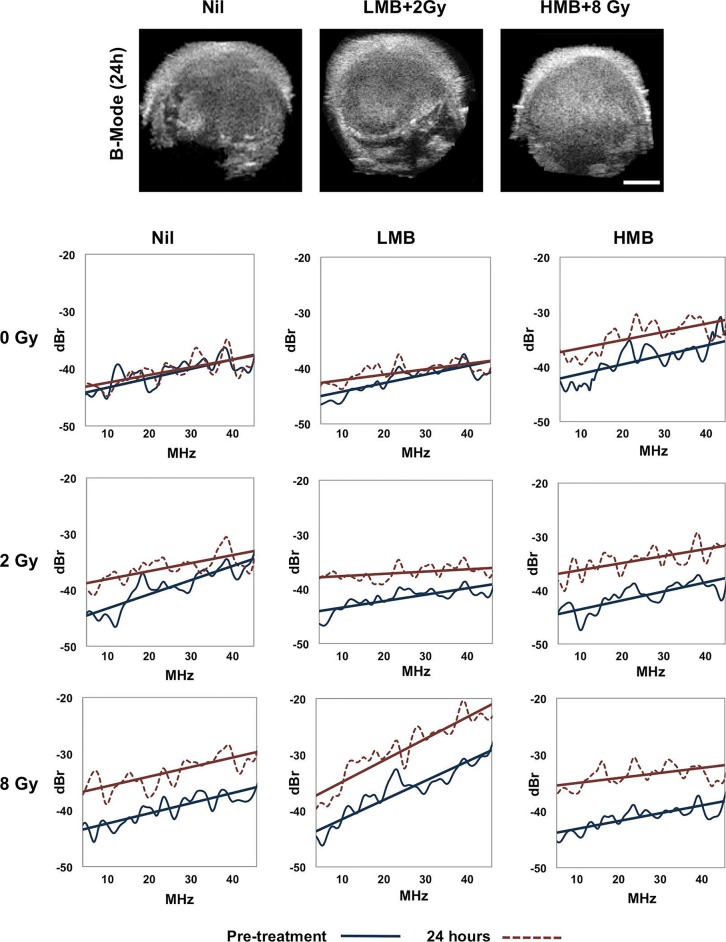
Representative B-Mode US of tumors and power spectra of samples under treatment conditions Power spectrum analysis was conducted at baseline (pre-treatment) and 24 hours post treatment. Changes in power spectrum were observed in treatments where higher doses of ultrasound mediated microbubbles and radiation were administered. For spectral parameters, the −6 dB window corresponded to a frequency range approximately 13-35 MHz. *Red line = Post-treatment (24h), Blue line = Pre-treatment, US Scale bar = 2mm, Nil= no microbubbles, LMB= Low microbubble concentration (1% v/v), HMB=High microbubble concentration (3% v/v)*.

Tumor response was monitored in this study using both non-invasive US imaging methods and immunohistochemistry. Representative B-mode images and power spectra are presented for samples in Figure 1. With increasing combined doses of microbubbles (LMB, HMB) and radiation (2 Gy, 8Gy), there was an increase in the spectral backscatter intensity in treated tumors compared to the control group (*p<0.05*). Summary changes of QUS parameters, and immunohistochemistry are described below.

### Mid-band fit (MBF)

**Figure 2:**
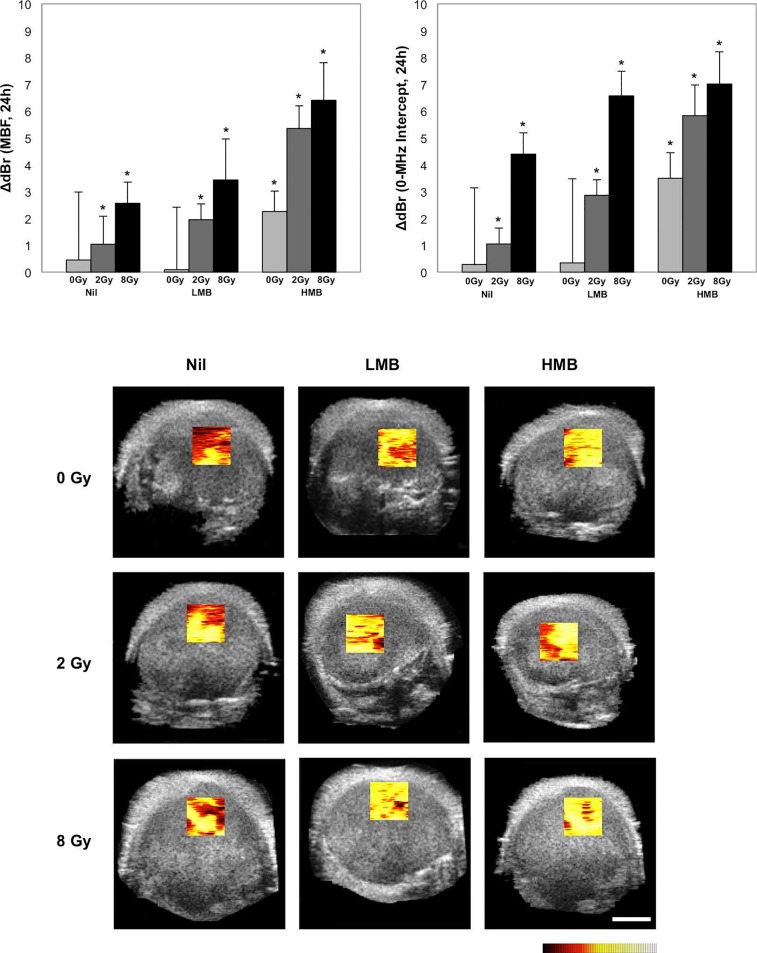
Changes in quantitative ultrasound parameters with treatment and corresponding parametric maps of the mid-band fit Significant increases in the mid-band fit (MBF) and 0-MHz intercept (SI) were observed in higher doses of ultrasound microbubbles and radiation, corresponding to central locations in the tumor. Tumors were treated with single doses of radiation (0 Gy, 2 Gy, 8 Gy) or microbubbles ( Nil, LMB, and HMB) or a combination of both treatment modalities. *p<*0.05, when compared to pre-treatment values*. Color scale represents a range of 20 dBr. Scale bar = 2 mm. Factorial ANOVA demonstrated significant treatment effects (radiation, microbubbles, radiation*microbubbles) on both the MBF and SI (p<0.001).

Figure 2 presents changes in the mid-band fit [ΔdBr (MBF, 24h)] in correspondence to 9 treatment conditions consisting of combinations of ultrasound-mediated microbubbles and radiation. Additionally, representative parametric maps of the MBF 24 hours after treatment are displayed. In the control group (Nil + 0 Gy), the MBF did not show any significant changes after 24 hours (+0.45 ± 2.53 dBr [±SD]; *p=0.210*). For radiation treatments only, there was an increase in the MBF of +1.04 ± 1.05 dBr; *p<0.05* when 2 Gy of radiation was given alone. For 8 Gy-treated tumors, the increase in the MBF was +2.56 ± 0.79 dBr (*p<0.05*). Low concentration microbubble (LMB) treatments alone, did not have a significant affect on tumors; the change was 0.09 ± 2.33 dBr; *p=0.790*. However, when combined with 2 Gy of radiation (LMB + 2 Gy), a significant increase was observed and the MBF increased by +1.95 ± 0.58 dBr (*p<0.05*) and this was also observed in LMB + 8 Gy treated tumors (+3.44 ± 1.53 dBr, *p<0.05*). Very strong treatment effects were measured in the high concentration microbubble (HMB) treated group, as measured by the MBF. Tumors treated with HMB alone demonstrated a significant increase in the MBF 24 hours treated (+2.26 ± 0.76 dBr; *p<0.05*). With increasing combined doses of radiation (2 Gy and 8 Gy), the MBF increased by 5.36 ± 0.85 dBr (*p<0.05*) and 6.41 ± 1.40 dBr (*p<0.05*), respectively. A two-way factorial ANOVA demonstrated significant treatment effects on the MBF with radiation treatment alone (*F*_2,98_ = 223.10, *p<.001*), microbubble treatment alone (*F*_2,98_ = 207.46, *p<.001*), and combined microbubbles and radiation treatments (*F*_4,196_ = 9.59, *p<.001*).

### 0-MHz intercept (SI)

Summary data for the 0-MHz intercept is presented in Figure 2. Control tumors and those treated with LMB alone did not demonstrate any significant changes in the SI after 24 hours of treatment (*p>0.05*). Radiation treatments alone (2 Gy, 8 Gy) demonstrated an increase in the SI of +1.04 ± 0.59 dBr (*p<0.05*), and +4.39 ± 0.79 dBr (*p<0.05*), respectively. Tumors treated with combination treatments had an increase in the SI, 24 hours after treatment. LMB + 2 Gy had an increase in the SI of +2.86 ± 0.58 dBr (*p<0.05*) and LMB + 8 Gy treated tumors showed an SI increase of +6.57 ± 0.92 dBr (*p<0.05*). High microbubble treatments alone resulted in an increased SI of +3.49 ± 0.95 dBr (*p<0.05*) and combination treatments with 2 Gy and 8 Gy showed a greater increase of +5.83 ± 1.14 dBr (*p<0.05*), and +7.01 ± 1.20 dBr *p<0.05*), respectively. A two-way factorial ANOVA demonstrated significant treatment effects on the SI with radiation treatment alone (*F*_2,98_ = 184.07, *p<.001*), microbubble treatment alone (*F*_2,98_ = 293.77, *p<.001*), and combined microbubbles and radiation treatments (*F*_4,196_ = 15.53, *p<.001*).

### Histological analysis

**Figure 3:**
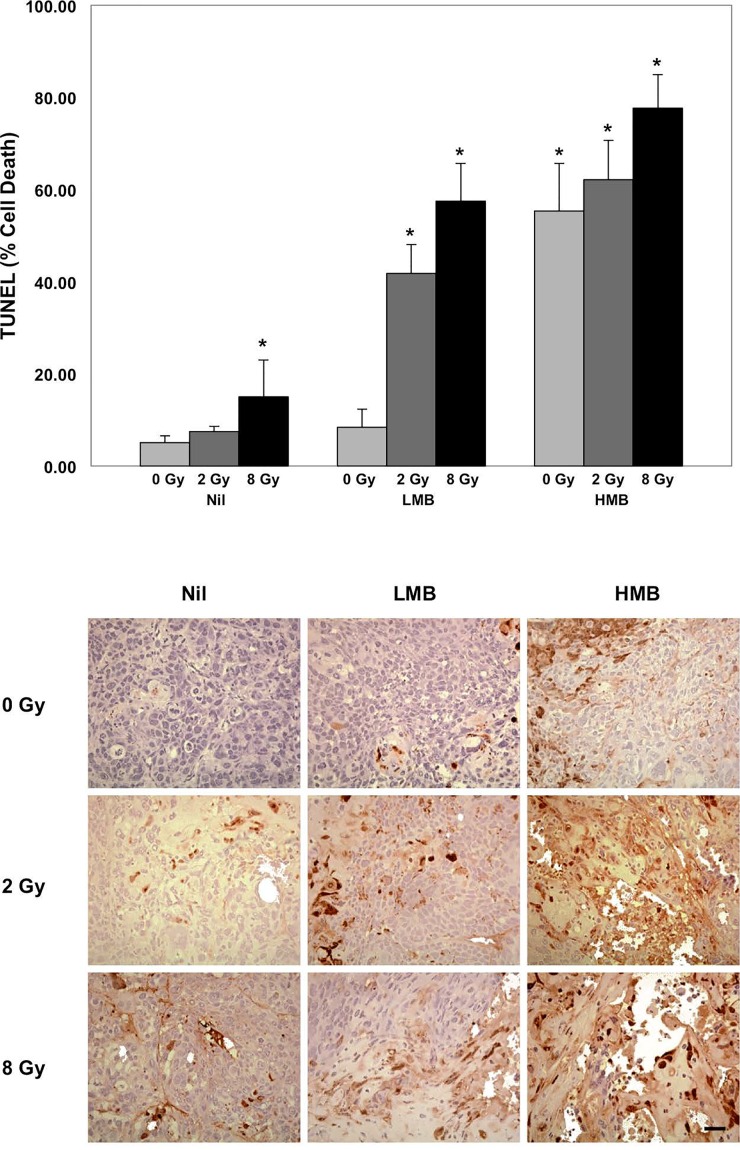
High magnification (TUNEL) staining at 24 hours after treatment and quantification of tumor cell death Treatment effects were observed in combination treatments. Increasing treatment doses resulted in higher areas of tumor cell death. *Histology*: Top Row. Microbubble treatments alone showed elevated levels of cell death with increased ultrasound-driven microbubbles treatment doses. Middle Row. 2 Gray radiation and Microbubble treatments. Additive effects are observed showing regions increased regions of cell death following higher doses of combination treatment. Bottom Row. Elevated regions of apoptosis as a result of microbubble treatment and radiation. Highest combination doses show areas of cell death and tumor cell death. Magnification=40x, Bar= 25 μm; *=p<0.05, compared to treatment controls (Nil + 0 Gy).

**Figure 4:**
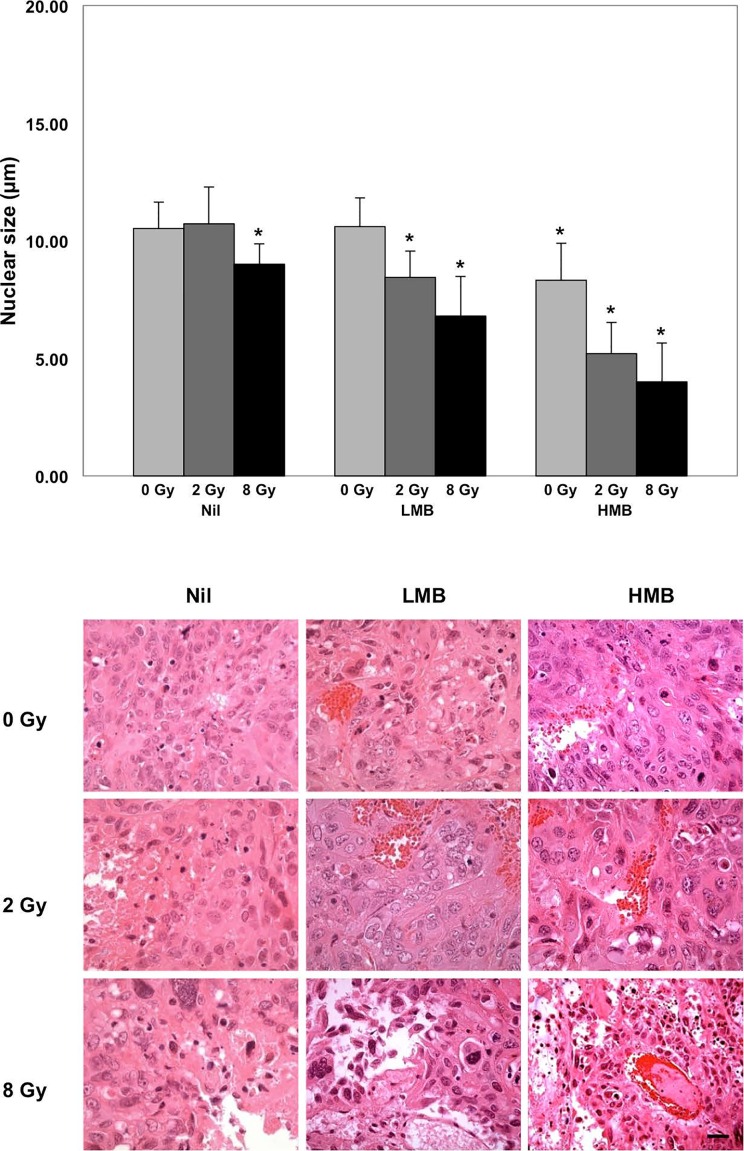
Hematoxylin and eosin staining, and nuclear size assessment Nuclear size was assessed in tumor cross-sections following 24 hours of treatment. Combination treatments demonstrated a significant difference in nuclear size compared to control samples (Nil + 0 Gy). *Histology*: Treatment from ultrasound-mediated microbubbles demonstrated vascular disruption, marked by areas with erythrocytes. Aggressive treatment doses showed a decrease in cellularity. Magnification=40x, Bar= 25 μm; *=p<0.05, compared to treatment controls (Nil + 0 Gy).

Immunohistochemical analyses are presented in Figure 3 and 4, and correspond to central regions of the tumor. Cell death analysis using TUNEL (Figure 3) showed that there were significant areas of cell death (% Cell Death) caused by treatment effects when 8 Gy of radiation was used to treat bladder cancer xenografts (*p<0.05*). A significant increase in cell death was also observed in combination treatment settings. The percentage of cell death was increased in tumors treated with high microbubble concentration (alone and in combination with radiation). Combinations of HMB + 8 Gy showed a %cell death of 77.67 ± 7.31 % (*p<0.05*). Treating tumors with HMB + 2 Gy also showed significant areas of cell death; these tumors showed a %cell death of 62.13 ± 8.48 % (*p<0.05*). High microbubble treatments alone showed an effect on tumor cell death, indicating a %cell death of 55.33 ± 10.33 % (*p<0.05*). Treatments with low microbubble concentrations also demonstrated significant areas of cell death. When tumors were treated with LMB and 2 Gy or 8 Gy, there was a % cell death of 41.80 ± 6.21 (*p<0.05*), and 57.47 ± 8.16 % (*p<0.05*), respectively. Control animals and those treated with LMB alone did not demonstrate any significant levels of cell death within tumor sections *(p>0.05)*. TUNEL staining and summary of data are presented in Figure 3.

Nuclear size was assessed, and quantitative data is presented in Figure 4. Hematoxylin and eosin staining of the tumor are also presented in Figure 4. Statistical analysis compared the mean nuclear size of treatment groups against the control (Nil + 0 Gy). Results indicated that tumors treated with 2 Gy only, or LMB only did not indicate statistically significant differences in the nuclear size compared to the control group (*p>0.05*). However, LMB-treated tumors showed significant differences when treated with 2 Gy or 8 Gy (*p<0.05*). Also, there were statistically significant differences in nuclear size with tumors treated with HMB + 0 Gy, or 2 Gy, or 8 Gy (*p<0.05*). The mean nuclear size was smallest in the HMB + 8 Gy group (4.00 ± 1.66 μm). There was also a reduction in the average nuclear size when tumors were treated with HMB alone, or combined with 2 Gy (8.32 ± 1.57 μm, and 5.20 ± 1.32 μm, respectively). LMB-treated tumors combined with 2 Gy or 8 Gy also showed a reduction in nuclear size (LMB + 2 Gy = 8.44 ± 1.12 μm; LMB + 8 Gy = 6.80 ± 1.68 μm). Radiation treatment alone, also had an effect on the tumor cells' nuclear size; 8 Gy treatments without microbubbles showed a mean nuclear size of 9.00 ± 0.87 μm. Hematoxylin and eosin staining showed areas of ischemia within the tumor, as well as areas of vascular disruption indicated by red blood cells within the tumor stroma (Figure 4).

## DISCUSSION

In the present study, we explored QUS for combination treatments using ultrasound-mediated microbubbles and radiation in bladder cancer xenografts. QUS was used to detect early changes in cellular and tissue features occurring in response to treatment. The current study highlights the potential role for definitive adjuvant radiotherapy and ultrasound-activated microbubbles for bladder cancer treatment; in addition to using QUS parameters as acute markers for response to these additive treatment effects *in vivo*. The results suggest that combining modalities can induce cell death and that QUS may be used to detect relevant and early markers that are sensitive to microscopic changes in bladder tumors. The implications for these applications could improve current radiotherapy treatments and help guide optimal therapy.

**Figure 5:**
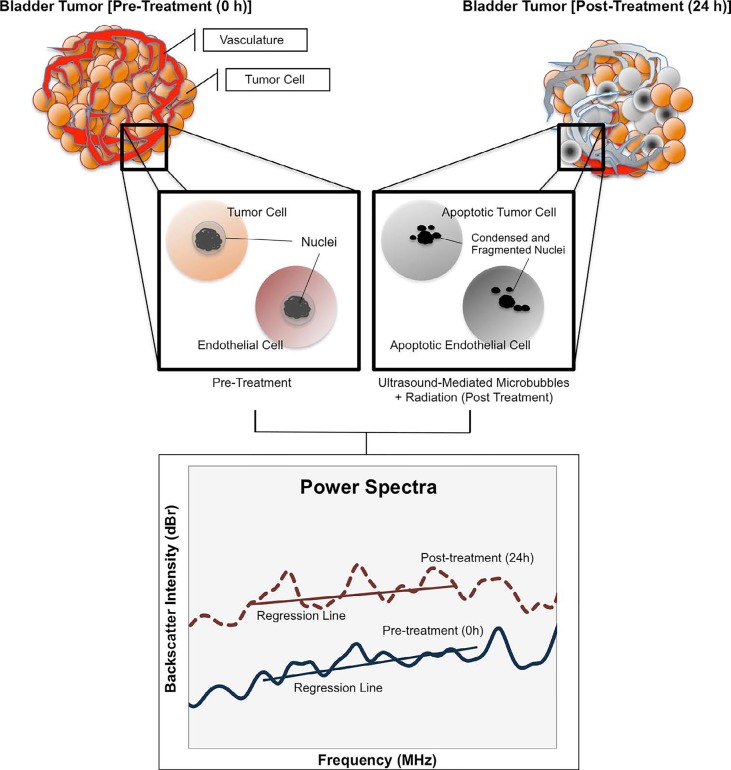
Schematic representation of QUS analysis in bladder cancer xenografts Bladder tumors were imaged prior to, and after 24 hours of treatment with ultrasound mediated microbubbles and radiation. Vascular disruption from microbubble cavitation resulted in endothelial cell death (apoptosis) followed by radiation-induced cell death in tumor cells. Spectrum analysis of tumors after 24 hours demonstrate an increase in the backscatter intensity likely caused by fragmented and condensed nuclear structures from both tumor cells and endothelial cells.

Previous reports have used both low and high (>20 MHz) frequency ultrasound to detect cell death from various treatments using spectral analysis [9, 10, 19-21, 26]. When exposed to cytotoxic agents, dying cells exhibit unique nuclear features involving pyknosis and karyorhexis. Structural formations such as nuclear condensation, and fragmentation affect the acoustical propagation in tissue and thus the backscatter intensity [21]. Lee *et al*. studied ultrasound-stimulated microbubbles and radiation treatments in prostate xenografts [9]. The maximum percentage of cell death in treated tumors was 63 ± 5%, corresponding to an increase in the mid-band fit and 0-MHz Intercept of 5.2 ± 1.4 dBr and 6.3 ± 1.9 dBr, respectively. Another study by Kim *et al*. showed the relationship between cell fragmentation and QUS; demonstrating a 17-fold increase in cell fragmentation relative to a change in the mid-band fit and 0-MHz Intercept (7.0 ± 4.1 dBr and 15.4 ± 2.5 dBr, respectively) [7]. In photodynamic-treated (PDT) melanoma treatments, Banihashemi and colleagues used high frequency ultrasound (26 MHz) to detect apoptotic cell death, and showed a statistically significant correlation between QUS changes and cell death up to 24 hours after PDT treatment [10]. Maximal increases in the MBF were observed after 12 hours, corresponding to an increase of 9.2 dBr [10]. In comparison to those studies, bladder cancer xenografts in this study demonstrated similar increases in both the MBF and SI at similar time intervals. Possible explanations for the obtained backscatter signal could be due to Rayleigh, and Mie scattering in tumors. Given the frequency (*f*) used (*f*=25 MHz), where the backscatter intensity (*I*) is proportional to *f ^4^*; and thus, dependent on wavelength (*I* ì *λ^−4^*), Rayleigh scattering predominates in very small particles relative to the wavelength, which could also include DNA fragments caused by cell death. In simulated models, smaller nuclear fragments have been predicted to depress the signal amplitude [27]. Alternatively, Mie scattering predominates for structures within the order of the ultrasound wavelength. It is possible that apoptotic cell clusters, nuclear coalescence and condensation, and larger hematomas (∼10-50 μm) in and around dying tumor cells exhibited Mie scattering in comparison to the cellular background. These biological factors may have contributed to an increase in the signal intensity [10]. Also, measurements of the backscatter intensity are further complicated by other tumor features such as, tumor water content (interstitial fluid), random distribution of scatterers, and particle size and concentration [18, 27]. Theoretical frameworks for spectrum analysis proposed by Lizzi *et al.* [18] showed that increases in the scatterers' diameters may also cause increases in the spectral intercept. In our histological analysis, there were observed patchy areas of aggregated erythrocytes in the intercellular space following ultrasound-microbubble treatment and radiation, and propose that these microscopic hematomas may have contributed to the increase in the spectral intercept (Figure 4). Increases in the mid-band fit could be explained as a result of changes in nuclear scattering from increased cell death. Although the nuclear size decreases with more aggressive treatment doses (Figure 5), the mechanical features such as particle density and the number (concentration) of scatterers across the tumor may have contributed to the increase in the MBF.

Experimental frameworks by Kolios *et al*. [20], and Czarnota *et al*. [19], have indicated that ultrasound backscatter signal amplitudes are linked to apoptosis. Indeed, other forms of cell death such as necrosis and oncosis are exhibited in tumors during therapy response, and these modes of cell death have been shown to alter the backscatter intensity based on scatterer randomization and distribution [18, 19, 21]. These processes highlight the tumor's heterogenic microenvironment, which is also affected by tumor cell assembly, stroma formation (i.e. collagen crosslinking), and the tumor's vascular architecture. Notably, the tumor's vascular plexus is tortuous, and composed of continuous layers of endothelial cells. Al-Mahrouki and colleagues previously showed that the endothelial cells were susceptible bystanders to mechanical stress from ultrasound stimulated microbubbles [28]. It was previously shown that endothelial cells' plasma membrane during microbubble cavitation initiated a ceramide-dependent pathway that committed cells to apoptosis [28]. That study reported that both tumor cells and endothelial cells were likely disposed for apoptotic cell death following microbubble-vascular disruption and radiation. It is proposed here, that the measured backscatter intensity were in part, caused by contributions in nuclear scattering from apoptotic tumor and endothelial cells, given the increase in backscatter intensity with tumors treated with both microbubbles and radiation (Figure 5).

Our study presents data for the first time, the use of QUS to detect cellular and subcellular changes in bladder tumors responding to ultrasound-mediated microbubbles and radiation. However, several other imaging studies are currently underway to obtain useful imaging biomarkers that predict tumor response to cytotoxic and targeted therapies [29-31]. Bailey *et al*. recently used dynamic contrast-enhanced MRI (DCE-MRI) to study *T_1_* and *T_2_* relaxation data for treatment monitoring [29]. Acute myeloid leukemia (AML) cells were treated with Cisplatin and showed a significant decrease in DCE-MRI parameters such as intracellular relaxation time, intracellular water fraction and an increase in the transmembrane water exchange rate in responsive AML cells. These results correlated with histological data suggesting a link to apoptosis [29]. Other studies have used radiolabelled markers, such as ^99m^Tc Annexin V *in vitro* and *in vivo* [30, 31]. Mochizuki *et al.* examined radiopharmaceutical uptake of ^99m^Tc Annexin V in hepatoma xenografts after treatment with Cyclophosphamide [31]. The authors concluded with data showing a significant increase in both apoptotic tumor cells (*p<0.001*) and the tumor cell uptake of ^99m^Tc Annexin V (*p<0.0001*).

In terms of imaging translation, QUS imaging could facilitate the development of responseguided therapies and develop adaptive radiotherapy plans according to tissue-response maps in bladder tumors. QUS parameters demonstrated sensitivity to microstructural changes in tissue from treatment effects Ultrasound-mediated microbubbles combined with radiation treatments could facilitate normal-tissue sparing by using targeted microbubble disruption of the tumor vasculature in combination with conformal radiotherapy. In principle, this could potentially be delivered through focused ultrasound for bubble stimulation, in addition to planning highly conformal dose constraints to bladder tumors *in vivo*. These treatments may offer a potential treatment option with curative intent in cases where cystectomy is contraindicated. We previously reported the longitudinal treatment effects in bladder cancer using microbubble vascular disrupting agents and radiation. The results of our previous study showed a significant decline in tumor survival in terms of tumor growth delay, and a reduction in vascular density, which was assessed histologically and by using power Doppler ultrasound [5]. Data from this present study complement our previous findings, and are consistent with other studies that demonstrated tumor-killing effects from both vascular disrupting agents and radiation concomitantly [4, 32-34]. Our study corroborates these other reports with findings to suggest:

1. Ultrasound-mediated microbubbles combined with radiation demonstrate an increase in backscatter intensity in bladder cancer xenografts, which is linked to treatment-induced apoptosis.
2. The increased backscatter intensity may be explained as nuclear pyknosis and karyorhexis from apoptosis. The distribution, concentration and randomization of nuclear fragments and condensed nuclear chromatin contribute to the backscatter signal detected by QUS in bladder tumors [27].
3. Bladder cancer tumors exhibit similar orders of magnitude in the change in backscatter intensity in comparison to other tumor models, such as prostate cancer when treated with combined ultrasoundmediated microbubbles and radiation [9]. The increase in backscatter intensity is relative to increases in doses given in combination therapies.

## MATERIALS AND METHODS

### Bladder cancer cell culture and animal xenograft model

Human bladder carcinoma HT-1376 cell lines (American Type Culture Collection, Manassas VA) were cultured in Eagle's Minimum Essential Medium (ATCC, Manassas VA) supplemented with 10% foetal bovine serum (Sigma Aldrich, St. Louis, Missouri, United States), 1% penicillin/streptomycin (Sigma Aldrich, St. Louis, Missouri, United States) and exposed to 5% CO_2_ HEPAfiltered air at 37°C. Cells were cultured and collected by adding 0.25% trypsin, 0.02% EDTA solution, washed with D-PBS and re-suspended in 100 μL D-PBS (Mg-, Ca-) per 1.0 *00D7* 10^6^ cells for injection. White-haired CB- 17 severe combined immuno-deficient (SCID) male mice (Charles River Inc., Wilmington, MA, USA) were injected subcutaneously with the cell suspension in the lower right hind leg. Tumors developed over a period of 2-3 weeks and measured 5-7mm at the time of experiments.

### *In vivo* treatment parameters

The institution's animal care committee approved the protocol and all procedures were performed under institutional research and ethical animal guidelines to mitigate animal suffering. A total of 45 animals were included in this study using a combination of treatments (9 treatment conditions; described below). Five animals were studied per treatment condition.

### Ultrasound-activated microbubble treatments

Definity microbubbles (Lantheus Medical Imaging, N. Billerica MA, USA) were generated by shaking, using a Lantheus vial mix device for 45 seconds at 3000 rpm. Three concentrations of microbubbles were used based on total mouse blood volume: No (nil), low (1% v/v, LMB) and high (3% v/v, HMB) [5, 6]. The microbubbles were diluted in sterile normal saline and injected into the mouse tail vein. An injection (0.1 cc) of normal saline was used to flush the tail vein prior to treatment. Mice were placed onto a custom-built mounting device, and the hind leg was immersed in a 37°C water bath for ultrasound coupling. Components of the ultrasound therapy system included a micropositioning system, waveform generator (AWG520, Tektronix, Beaverton USA), power amplifier with pulser/receiver (RPR4000, Ritec, Warwick USA), and a digital acquisition system (Acquiris CC103). Treatment parameters were previously optimized by Karshafian *et al*. [24] and adapted for this study. Briefly, the tumors were exposed within the half peak maximum of the acoustic signal (−6 dB beam width of 31 mm and depth of field greater than 2 cm) pulsed at 500kHz center frequency using a 2.85 cm unfocused planar ultrasound transducer (Valpey Fisher Inc, Hopkinton USA). Ultrasound exposure comprised of tone bursts (16 cycles), 3 kHz pulse repetition frequency for 50 ms. The peak negative pressure was set to 570kPa, which corresponded to a mechanical index (MI) of 0.8. An intermittent 1950 ms period between bubble sonications was employed to mitigate biological heating during ultrasound exposures. The total insonification time was 750 ms over 5 minutes.

### Single fraction radiation treatment

Immediately after microbubble treatment, mice were transferred for tumor irradiation using an irradiation cabinet device (Faxitron, Wheeling Illinois, USA). Single fraction doses of 0, 2 or 8 Gy were administered at a dose rate of 200 cGy/minute, using 160 kVp energy and at a source-skin distance (SSD) of 30 cm. Lead shielding was used with an open aperture in order to focus radiation treatments to the tumor only.

### *In vivo* data acquisition and analysis

Mice were anaesthetized with 2% oxygen ventilated isoflurane for induction and then treated with an intraperitoneal injection of ketamine 100 mg/kg, xylazine 5mg/kg, and acepromazine 1mg/kg in 0.1 mL saline solution (0.9% sodium chloride) for anaesthetic continuance during treatments. Anaesthetic administration was titrated within 0.02 mL increments to optimize mouse survival. All mice were monitored to maintain standard temperature, heart rate and respiration. Animals were imaged before treatments (baseline) in order to determine pre-treatment tumor characteristics and again 24 hours after treatment to characterize treatment response. Standard B-mode and raw radiofrequency (RF) data was acquired using a VEVO770 ultrasound unit (VisualSonics, Toronto Canada) with a 25 MHz transducer (VisualSonics RMV-710B, lateral resolution=149 μm, axial resolution=54 μm). Data collection acquired typically 60-80 frames in a 3D volumetric scan mode for both B-mode images and RF data. The center of the tumor was positioned at the acoustic focus and data was collected at a sampling frequency of 420 MHz.

Analysis of ultrasound RF data was conducted by calculating a normalized power spectrum. This analysis was performed by selecting rectangular regions of interest (ROI) from 10 scan planes within the tumor. ROIs were selected in the centre of the tumor accounting for approximately 2/3 of the tumor cross sectional area (approximately 5-10 × 5-10 mm in-plane and 5-10 mm through plane). Spectral parameters were computed using a sliding window analysis method within each ROI. A gated RF window with 90% overlap between adjacent windows in the axial direction was used. Data was normalized using a calibration pulse collected on the same ultrasound system and transducer using a tissue equivalent phantom. The phantom was composed of glass microspheres (5-40 μm) in order to remove system transfer function effects by spectral subtraction. This normalization process calibrates the sample spectra by accounting for differences in the ultrasound signal such as focus and beam forming effects.

Spectral parameters were determined by applying a linear regression across a −6 dB window taken from the peak in the normalized power spectrum [19]. This corresponded to a frequency range of approximately 13 MHz to 35 MHz. QUS parameters were collected at each frame (10 frames per animal/tumor) and spectral data were averaged across the ultrasound scan. Parameters extracted from the RF dataset included the mid-band fit (MBF), and 0-MHz intercept (SI), chosen based on their close relationship with acoustic backscatter and scatterer property information such as size, shape and concentration [12]. Principles describing these parameters have been comprehensively described elsewhere [14, 20, 24, 25].

### Histopathology

After 24 hours, tumors were excised and fixed in 10% acetate buffered formalin (Fisher Scientific Canada, Ottawa Ontario, Canada). Fixation was carried out at room temperature for 4 hours and then samples were transferred to 4°C for 24 hours. Whole-tumor samples were then processed using a Leica ASP300 smart tissue processor (Leica Microsystems, Richmond Hill, Ontario, Canada). Samples were prepared in paraffin blocks (Leica EG 1160, Leica Microsystems, Richmond Hill, Ontario, Canada) and sectioned into 5 μm for slide preparation. Tissues were stained with standard hematoxylin and eosin (H&E) techniques. To visualize cell death, additional slides were prepared for TdT-mediated dUTP-biotin nickend-labeling (TUNEL). Specimens were analyzed by microscopy at high magnification (40x) and tumor cross sections were subsequently digitized. Specimen analysis was performed using microscopy and imaging software (ImageJ, NIH, Bethesda, Maryland, USA). Cell death fraction (% cell death) was assessed by first selecting a region of interest (ROI) spanning the entire cross-sectional area of the tumor specimen. For each mouse, 3 sections were analyzed and averaged. The percentage of cell death (% cell death), was calculated as the ratio between positively-stained areas of cell death to the total cross sectional tumor area. Cell nuclear size as also assessed digitally (ImageJ, NIH, Bethesda USA), adapted from methods previously described by Banihashemi *et al.* [10]. Briefly, the mean nuclear size was obtained by analyzing 5 ROIs per mouse within tumor cross sections.

### Statistical analysis

Statistical analysis was performed using SPSS (IBM Corp, Armonk USA), using all frames analyzed within each treatment condition (n=50 frames/treatment condition). A paired *t-test* was used to compare for significant differences in QUS parameters, nuclear size and cell death (TUNEL) after 24 hours. An alpha level of 0.05 or less was considered significant. Additionally, a two-way factorial analysis of variance (ANOVA) was utilized to test for significant treatment effects on QUS parameters, corresponding to the F-ratios.

## CONCLUSION

The current study demonstrates the use of quantitative ultrasound to monitor tumor response to ultrasound-stimulated microbubbles and radiation in bladder cancer xenografts, non-invasively. The therapy approach examined here has potential applications in radiation oncology and with further studies, ultrasoundmediated microbubbles may also be used to optimize radiation doses through radiosensitization of tumors. This treatment approach may potentially be used for personalized treatments that combine conformal radiation plans with focused ultrasound activation at the tumor site. Additionally, ultrasound spectrum analysis could be used to guide therapy, and also verify treatment efficacy *in vivo*.
